# Antibody Boosting and Longevity Following Tetanus Immunization During Pregnancy

**DOI:** 10.1093/cid/cis979

**Published:** 2013-03-01

**Authors:** Freya J. I. Fowkes, Rose McGready, Simon Johnstone-Robertson, François Nosten, James G. Beeson

**Affiliations:** 1 Macfarlane Burnet Institute of Medical Research; 2 Centre for Molecular, Environmental, Genetic and Analytic Epidemiology, Melbourne School of Population Health, University of Melbourne; 3 Department of Epidemiology and Preventive Medicine, Monash University, Melbourne, Australia; 4 Shoklo Malaria Research Unit, Mae Sot, Tak; 5 Faculty of Tropical Medicine, Mahidol University, Bangkok, Thailand; 6 Centre for Tropical Medicine, Nuffield Department of Clinical Medicine, University of Oxford, CCVTM, United Kingdom; 7 Department of Microbiology, Monash University, Melbourne, Australia

To the Editor—Maternal and neonatal tetanus is a significant cause of mortality, estimated to cause 180 000 deaths annually [1]. Since the mid-1970s, tetanus vaccination of pregnant women has been included in the World Health Organization's (WHO) Expanded Programme on Immunisation (EPI) [2]. Two doses of tetanus toxoid are sufficient to generate an antibody response (immunoglobulin G [IgG] class) capable of protecting neonates from tetanus, 3 doses are recommended for pregnancy, and 5 are recommended for life [3]. Despite these recommendations, WHO has identified a lack of longitudinal data quantifying antitetanus antibody boosting and duration during pregnancy following immunization in the EPI schedule [4].

To address this gap, we determined levels of antitetanus IgG at multiple time points from enrollment to delivery (median, 30 weeks of follow-up) in 376 pregnant women participating in malarial antibody studies at the antenatal clinics of the Shoklo Malaria Research Unit in northwest Thailand (previously published with ethics statement in Fowkes et al [5]). The tetanus vaccination regimen (tetanus toxoid) followed EPI guidelines [3]: dose 1, as early as possible during pregnancy; dose 2, one month after dose 1; dose 3, 6 months after dose 2; dose 4, 1 year after dose 3; and dose 5, one year after dose 4. During the study, 48.9% of women received their first dose, 86.2% received doses 2–4, and 8.2% received the final dose (dose 5).

**Figure 1.**
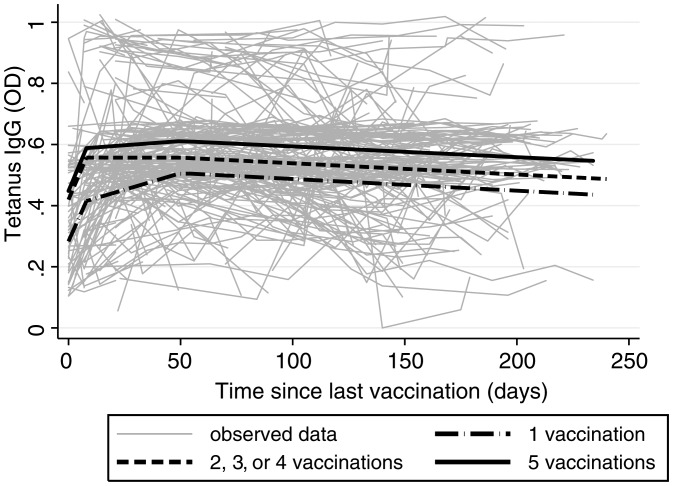
Antitetanus immunoglobulin G (IgG) after vaccination in 376 pregnant women according to vaccination dose. Tetanus IgG levels (optical density) were determined by enzyme-linked immunosorbent assay as previously described [5] with tetanus toxoid coated at 0.4 colony-forming units/mL and sera tested at a 1:500 dilution. Lines represent predicted mean tetanus IgG levels (calculated by mixed linear models with random effect for the intercept, slope, and covariance). The best-fit model had linear splines placed at 8 and 50 days, and each vaccination category had its own slope for antibody level over time since vaccination. Analysis was unadjusted because other potential confounders including gravidity, trimester, chloroquine prophylaxis [7], and *Plasmodium* species infection [8] did not significantly alter the model outputs. Abbreviations: IgG, immunoglobulin G; OD, optical density.

The boosting and decay of tetanus antibody levels after vaccination was vaccine dose-dependent (Figure 1). In the first 8 days after vaccination, antitetanus IgG increased rapidly at comparable rates in all vaccination groups (*P* > .85 relative to T1). Interestingly, at 8 days after vaccination, IgG responses peaked and then plateaued in those receiving ≥2 doses. In contrast, IgG responses in those receiving their first vaccination peaked later at 50 days after vaccination (Figure 1, *P* < .001). After 50 days postvaccination, antitetanus IgG responses declined and calculated IgG half-life was dependent on vaccination dose: 7.12 years (95% confidence interval [CI], 3.02–∞) for dose 1; 10.97 years (6.71–∞) for doses 2–4; and 12.28 years (6.15–∞) for dose 5. These estimates are in concordance with published nondose-specific half-life estimates in nonpregnant American women (10 years, 95% CI, 8–14) [6].

The close consecutive sampling of antitetanus antibody levels has allowed us to define, in the greatest detail to date, antitetanus IgG kinetics postvaccination, and we provide the first estimates of tetanus IgG half-lives in pregnancy according to vaccination dose in the EPI schedule. These data are important for predicting protection in neonates and are invaluable for understanding the sustainability of protective humoral immunity in high-risk populations such as pregnant women in resource-poor settings.
